# Genome-wide association study of nocturnal blood pressure dipping in hypertensive patients

**DOI:** 10.1186/s12881-018-0624-7

**Published:** 2018-07-04

**Authors:** Jenni M. Rimpelä, Ilkka H. Pörsti, Antti Jula, Terho Lehtimäki, Teemu J. Niiranen, Lasse Oikarinen, Kimmo Porthan, Antti Tikkakoski, Juha Virolainen, Kimmo K. Kontula, Timo P. Hiltunen

**Affiliations:** 1 Department of Medicine, University of Helsinki and Helsinki University Hospital, 00290 Helsinki, Finland; 2 Faculty of Medicine and Life Sciences, University of Tampere and Tampere University Hospital, Tampere, Finland; 3 National Institute for Health and Welfare (THL), Helsinki, Finland; 4 Department of Clinical Chemistry, Fimlab Laboratories and Finnish Cardiovascular Research Center Tampere, Faculty of Medicine and Life Sciences, University of Tampere, Tampere, Finland; 5 National Heart, Lung, and Blood Institute’s and Boston University’s Framingham Heart Study, Framingham, MA USA; 6 Division of Cardiology, Heart and Lung Center, University of Helsinki and Helsinki University Hospital, Helsinki, Finland

**Keywords:** Blood pressure dipping, Genome-wide, Circadian gene, BCL11B, ERAP2, Left ventricular hypertrophy

## Abstract

**Background:**

Reduced nocturnal fall (non-dipping) of blood pressure (BP) is a predictor of cardiovascular target organ damage. No genome-wide association studies (GWAS) on BP dipping have been previously reported.

**Methods:**

To study genetic variation affecting BP dipping, we conducted a GWAS in Genetics of Drug Responsiveness in Essential Hypertension (GENRES) cohort (*n* = 204) using the mean night-to-day BP ratio from up to four ambulatory BP recordings conducted on placebo. Associations with *P* < 1 × 10^− 5^ were further tested in two independent cohorts: Haemodynamics in Primary and Secondary Hypertension (DYNAMIC) (*n* = 183) and Dietary, Lifestyle and Genetic determinants of Obesity and Metabolic Syndrome (DILGOM) (*n* = 180). We also tested the genome-wide significant single nucleotide polymorphism (SNP) for association with left ventricular hypertrophy in GENRES.

**Results:**

In GENRES GWAS, rs4905794 near *BCL11B* achieved genome-wide significance (*β* = − 4.8%, *P* = 9.6 × 10^− 9^ for systolic and *β* = − 4.3%, *P* = 2.2 × 10^− 6^ for diastolic night-to-day BP ratio). Seven additional SNPs in five loci had *P* values < 1 × 10^− 5^. The association of rs4905794 did not significantly replicate, even though in DYNAMIC the effect was in the same direction (*β* = − 0.8%, *P* = 0.4 for systolic and *β* = − 1.6%, *P* = 0.13 for diastolic night-to-day BP ratio). In GENRES, the associations remained significant even during administration of four different antihypertensive drugs. In separate analysis in GENRES, rs4905794 was associated with echocardiographic left ventricular mass (*β* = − 7.6 g/m^2^, *P* = 0.02).

**Conclusions:**

rs4905794 near *BCL11B* showed evidence for association with nocturnal BP dipping. It also associated with left ventricular mass in GENRES. Combined with earlier data, our results provide support to the idea that *BCL11B* could play a role in cardiovascular pathophysiology.

**Electronic supplementary material:**

The online version of this article (10.1186/s12881-018-0624-7) contains supplementary material, which is available to authorized users.

## Background

Blood pressure (BP) follows a diurnal pattern and is generally lower at night than during the day, defined as BP dipping [1]. Attenuated nocturnal BP dipping has been associated with cardiovascular target organ damage and increased risk for cardiovascular events [2, 3]. Recent meta-analysis, examining the prognostic significance of nocturnal systolic BP fall in 17,312 hypertensives from three continents, demonstrated that blunted nighttime BP dipping in untreated hypertensive subjects predicted significantly a variety of cardiovascular end points, including coronary events, strokes, cardiovascular deaths and total deaths [4]. This risk increase was found to be independent of ambulatory 24-h BP levels [4]. In fact, international guidelines for hypertension recommend routine use of 24-h ambulatory BP measurement (ABPM) to assess the nighttime BP and BP dipping [5]. However, the cause of variation in BP dipping is not well established. Better understanding of the phenomenon could help identifying those with increased risk for cardiovascular morbidity and mortality.

Large scale genome-wide association studies (GWAS) have identified several susceptibility loci for elevated BP, which together explain only a few percent of the trait variance suggesting that many causal loci remain unidentified (for review, see [6]). It is thought that improving accuracy of BP measurements as well as introducing new phenotypic parameters that better correlate to cardiovascular risk could lead to discovery of new genetic loci [6]. Like BP levels, the BP dipping status may be partly inherited; Wang et al. [7] reported that 59% of the heritability in systolic BP (SBP) dipping and 81% of the heritability in diastolic BP (DBP) dipping were due to genetic influence, arousing interest to find the genes that affect BP dipping.

In animal studies, mutation or deletion of core circadian genes operating the internal clock resulted in disruption of the circadian variation of BP [8, 9]. This hypothesis was tested in two candidate gene studies on Taiwanese [10] and Chinese [11] populations that reported suggestive associations between certain circadian gene polymorphisms and BP dipping encouraging further research for their role. A different approach was taken by Wirtwein et al., who recently found that five risk loci for coronary artery disease (CAD), derived from GWASs, were also associated with non-dipping status in patients with CAD [12]. However, no GWASs on BP dipping have been before reported.

We have previously conducted a GWAS on antihypertensive drug responsiveness in the Genetics of Drug Responsiveness in Essential Hypertension (GENRES) study [13], with replication of the data in Finnish participants of the Losartan Intervention for Endpoint Reduction in Hypertension (LIFE) study [14]. A special feature of the GENRES study was the inclusion of four 4-week drug-free placebo periods in its design [15], generating a most useful opportunity to obtain repeated ABPMs and nocturnal BP dipping measurements for every participant. Here we report our GWAS study on BP dipping, mostly based on the GENRES study, but also containing replication data from Finnish patients of two other studies: Haemodynamics in Primary and Secondary Hypertension (DYNAMIC) [16, 17] and Dietary, Lifestyle and Genetic determinants of Obesity and Metabolic Syndrome (DILGOM) [18].

## Methods

### Discovery cohort – The GENRES platform

Rationale and results of the GENRES study (clinicaltrials.gov NCT03276598, registered retrospectively) have been previously published elsewhere [13–15]. In brief, GENRES is a double-blind, randomized, placebo-controlled cross-over study that was designed to search for genetic variation predicting response to four classes of antihypertensive drugs, conducted at Helsinki University Hospital between years 1999 and 2004 (Additional file 1: Figure S1). Initially, a total of 313 Finnish men (aged 35 to 60 years) with moderate hypertension (DBP ≥ 95 mmHg or previous use of antihypertensive medication) were screened for the study. Secondary hypertension, drug-treated diabetes mellitus, congestive heart failure and CAD were among the exclusion criteria. The study participants underwent 4 different one-month single-drug treatment periods (losartan 50 mg, amlodipine 5 mg, hydrochlorothiazide 25 mg, and bisoprolol 5 mg daily) separated by one-month placebo periods. Twenty-four-hour ABPM was carried out at the end of each drug and placebo period using a device equipped with a position sensor (Diasys Integra; Novacor, Rueil-Malmaison, France). Each subject gave written informed consent and the study was approved by the Ethics Committee of Helsinki University Central Hospital and National Agency of Medicines of Finland.

For the discovery GWAS, we used the ABPMs recorded at the end of each placebo period. BP readings were taken every 15 min when standing and every 30 min when supine. Intense physical activity was not permitted. Daytime was defined as hours between 7 am and 10 pm. Single observations were excluded from the analysis due to low pulse pressure (< 15 mmHg if SBP < 120 mmHg and < 20 mmHg if SBP > 120 mmHg), high heart rates (≥ 110 bpm), lying down during daytime, standing up or being awake at nighttime or high physical activity. For a recording to be accepted, > 15 daytime and > 7 nighttime measurements were required. The mean SBP and DBP dipping of all placebo periods were used as the study variables. A total of 235 unrelated subjects were successfully genotyped and had ABPM data from at least one placebo period. To reduce the effect of day-to-day variation in the dipping phenotype, only the 204 subjects that had ≥3 accepted placebo ABPMs available (173 subjects with four and 31 subjects with three ABPMs) were included in the discovery GWAS.

### Replication cohorts

DYNAMIC (clinicaltrials.gov NCT01742702) is an on-going study to investigate hemodynamic changes in primary and secondary hypertension with non-invasive hemodynamic measurement [16, 17]. A total 188 subjects with ABPM dipping available were genotyped. One subject was excluded due to low success rates of genotyping, one subject was excluded due to morbid obesity (body mass index (BMI) > 40) and 3 subjects were excluded due to first degree relativeness; ultimately, 183 subjects were included in this replication cohort. BP was measured with Microlife WatchBP O3 monitor (Microlife AG, Widnau, Switzerland) every 20 min during the day (7 am to 10 pm) and every 30 min during the night (10 pm to 7 am). Single observations were excluded using the same criteria as described for GENRES. Each subject gave written informed consent and the study was approved by the Ethics Committee of Tampere University Hospital.

DILGOM was a population survey conducted in 2007 to assess the effects of environment and genetics on obesity and metabolic syndrome. Four hundred and ninety-four subjects of DILGOM participated in a cardiovascular substudy. In 2014, 290 still living participants of the cardiovascular substudy underwent re-examination including a 24-h ABPM to compare novel and traditional BP measurement methods [18]. Both BP dipping and genetic data were available for 207 unrelated subjects. Four subjects that were morbidly obese (BMI > 40) and 23 subjects that were over the age of 75 were excluded, resulting in 180 subjects to be included in the replication cohort. BP was measured with a Microlife WatchBP O3 monitor (Microlife AG, Widnau, Switzerland) every 20 min during the day (7 am to 10 pm) and every 30 min during the night (10 pm to 7 am). Night-time BP was defined as the mean of all BP values of the actual sleeping period and daytime BP as the mean of all other BP values. Each subject gave written informed consent and the study was approved by the Ethics Committee of the Hospital District of Southwest Finland.

### Genotyping methods

The genotyping methods and quality control steps (including exclusions for identity-by-state clustering, gender check, Hardy-Weinberg equilibrium *P* value < 1 × 10^− 5^ and minor allele frequency < 0.01) for GENRES have been described in detail before [13]. The DNA samples of the GENRES study subjects were genotyped at the Institute for Molecular Medicine Finland (Helsinki, Finland) using the Illumina HumanOmniExpress BeadChip (Illumina, San Diego, CA, USA). After quality control steps a total of 631,844 autosomal SNPs were available for the analysis. Subsequent imputation was performed using IMPUTE2 [19] and the Hapmap2 CEU release 22 reference panel after pre-phasing with ShapeITv2. However, only the genotyped SNPs were used for the GWAS analysis.

The DNA samples of the DYNAMIC study subjects were genotyped at the Institute for Molecular Medicine Finland (Helsinki, Finland) using the Illumina HumanOmniExpress BeadChip (Illumina, San Diego, CA, USA). One subject was excluded due to low success rate (< 95%). The six SNPs used for replication passed the same quality control steps as described for GENRES above.

The genotype data of the DILGOM study subjects was derived from previously conducted GWASs. The DILGOM DNA samples were genotyped with Illumina Cardio-MetaboChip and Illumina 610 K arrays (Illumina, San Diego, CA, USA) at the Wellcome Trust Sanger Institute (Cambridge, UK) and the Institute for Molecular Medicine Finland (Helsinki, Finland). Unobserved SNPs were imputed using 1000 Genomes haplotypes Phase I integrated variant set release (ShapeITv2) in NCBI build 37 version June 2014. If the selected SNP was not genotyped, then the imputed value was used. Info scores for the imputed SNPs used were 0.989 for rs16984571 and 0.998 for rs1230361.

### Left ventricular hypertrophy in GENRES

Left ventricular hypertrophy (LVH) was assessed in GENRES from the transthoracic echocardiograms recorded during the first placebo period and from electrocardiograms (ECGs) recorded at the end of each placebo period [20]. Echocardiographically determined left ventricular mass (LVM, in grams) was calculated as 0.8 × [1.04 × ((interventricular septal thickness + left ventricular end-diastolic diameter + posterior wall thickness)^3^ – left ventricular end diastolic diameter^3^)] + 0.6 and left ventricle mass index (LVMI) by dividing LVM by body surface area (square meters). LVMI was available for 227 GENRES subjects that were successfully genotyped for rs4905794. The standard resting 12-lead ECG was recorded with Marquette MAC 5000 electrocardiograph (GE Marquette Medical Systems, Milwaukee, Wisconsin, USA) at the end of each placebo period and all measurements were performed blinded to all other data. Sokolow-Lyon voltage (SV1 + RV5 or SV1 + RV6, whichever was greater) and Cornell voltage-duration product [(SV3 + RaVL) × QRS duration] were used as markers of LVH. The indices were calculated from digital ECGs from automatically made ECG measurements with a visual confirmation for all subjects who had completed the whole study. Mean values of all placebo periods were used. A total of 185 subjects, that were successfully genotyped for rs4905794, had these ECG measurements available.

### Statistical analysis

Baseline demographics and BP dipping data of all study subjects were analyzed using IBM SPSS Statistics 22.0 (IBM Corp., Armonk, NY, USA). SBP and DBP dipping were analyzed separately as continuous variables, and were calculated as night-to-day BP ratio [3] and expressed as percentage [(mean nighttime BP / mean daytime BP) × 100]. Both variables were adjusted for corresponding daytime BP using linear regression. Lifestyle factors (age, BMI, waist-hip ratio, antihypertensive use before entry to the study, duration of hypertension, smoking, serum creatinine level and daily urinary excretion of sodium after the first placebo period were tested for association with stepwise linear regression and included as covariates if *P* value for association was < 0.10 for either systolic or diastolic night-to-day BP ratio. The final regression models are presented in Additional file 2: Table S1. These regression variables were approximately normally distributed. The effect of population stratification on night-to-day BP ratio was tested with principal components (PCs) generated with program Smartpca from Eigensoft package [21]. A reduced dataset of 114,487 SNPs with r^2^ < 0.5 and the long-range linkage disequilibrium (LD) regions excluded [22] was used. Three significant PCs for population stratification with *P* values < 0.10 were identified with program Twstats from Eigensoft package, but they were not associated with either systolic or diastolic night-to-day BP ratio residuals (all P values > 0.30) and were thus not included in GWAS analysis. The discovery GWAS was done using covariate-adjusted night-to-day BP ratio residuals and linear regression under additive genetic model in PLINK v1.07 [23]. *P* values < 5 × 10^− 8^ were considered as genome-wide-level significant, while *P* values < 1 × 10^− 5^ were considered as suggestively significant.

The statistically most significant SNPs from each locus with *P* value < 1 × 10^− 5^ were selected for replication in two independent Finnish cohorts. For the replication cohorts similar statistical approach was used. Bonferroni corrected *P* value < 0.004 was regarded as a successful replication [0.05 / (2 studies × 6 SNPs)], considering that systolic and diastolic dipping are highly correlated. *P* value < 0.05 with the same direction of effect was considered as suggestive replication. We also conducted meta-analyses of the top BP dipping (night-to-day BP ratio) SNPs in all available studies using inverse-variance model with fixed effects in METAL [24]. We defined significant results as *P* values < 5 × 10^− 8^. *P* values < 1 × 10^− 5^ were considered to represent a suggestive association.

In addition, we tested association of the genome-wide-significant SNP (rs4905794) with LVMI, Sokolow-Lyon voltage and Cornell voltage-duration product measured in GENRES subjects to further explore the nature of the SNP in relation to target organ damage. We tested for significant covariates and adjusted LVMI with age (standardized *β* = 3.3, *P* = 0.007) and Sokolow-Lyon voltage with BMI (standardized *β* = − 0.6, *P* = 0.0003) using stepwise linear regression (other covariates tested were smoking, waist-hip ratio and previous use of antihypertensive medication). BP levels were not included as covariates (although highly significant) as we presumed the effect of the genotypes to be principally mediated through BP levels. No covariates were significant for Cornell voltage-duration product.

A SNP set-based approach was used to test for association of circadian genes with BP dipping (night-to-day BP ratio). The 24 genes listed in the circadian rhythm pathway in KEGG (Kyoto Encyclopedia of Genes and Genomes) PATHWAY database [25, 26] were selected for testing. All successfully genotyped SNPs located inside the transcript boundaries of each circadian gene were included. Transcript boundaries of the circadian genes were derived from Ensembl Genome Browser (release 87, in NCBI37/hg19) [27, 28]. The set-based tests were conducted in PLINK v1.07 [23]. Using the SBP and DBP dipping (night-to-day BP ratio) residuals calculated as described above, a mean statistic was calculated for each gene based on the single-SNP association results under a linear model. Single SNPs were included in the set if association *P* value was < 0.05 and *r*^*2*^ was < 0.5 with other SNPs selected with maximum of five SNPs included for each gene. The dataset was then permuted 10,000 times calculating empirical *P* value for each gene describing the number of times the permuted set-statistic exceeded the original one for the gene. Bonferroni corrected *P* value of 0.002 (0.05/24) was used to correct for multiple testing (24 genes).

Finally, we sought to replicate the previously published genetic associations, based on candidate gene studies, with BP dipping. This included five SNPs from Leu et al. [10], two SNPs from Sheng et al. [11] (one of which is shared with Leu et al.) and five SNPs from Wirtwein et al. [12]. Two of these SNPs were not genotyped in GENRES and thus imputed values were used. We considered Bonferroni corrected *P* value < 0.0045 (0.05/11 SNPs tested) and the same direction of effect as successful replication and *P* value < 0.05 and the same direction of effect as suggestive replication.

### Functional analysis for top associations

We assessed the consequence of the leading SNPs of the loci that achieved *P* value < 1 × 10^− 5^, using VEP (variant effect predictor) database [29, 30]. To look for putative functional consequences, we annotated each SNP to the closest gene using Ensembl database (release 87) [27, 28] and conducted an expression quantitative trait locus (eQTL) analysis in publicly available Genotype Tissue Expression (GTEx) database [31, 32]. We then used PhenoScanner [33, 34] to find if any of the SNPs had previously reported cardiovascular trait associations at a nominal significance of *P* < 0.05.

The Neale lab has made available basic GWAS results for over 2000 phenotypes from the UK Biobank data of ~ 337,000 unrelated individuals of British ancestry [35]. We looked up our leading SNPs for BP dipping through MR-Base PheWAS database [36] to see if they were associated with SBP or DBP, or with seven sleep traits (sleep duration, getting up in morning, chronotype, nap during day, sleeplessness, snoring, daytime dozing) in the Neale lab data, at a nominal significance of *P* < 0.05.

## Results

### Genome-wide association study in GENRES

**Fig. 1:**
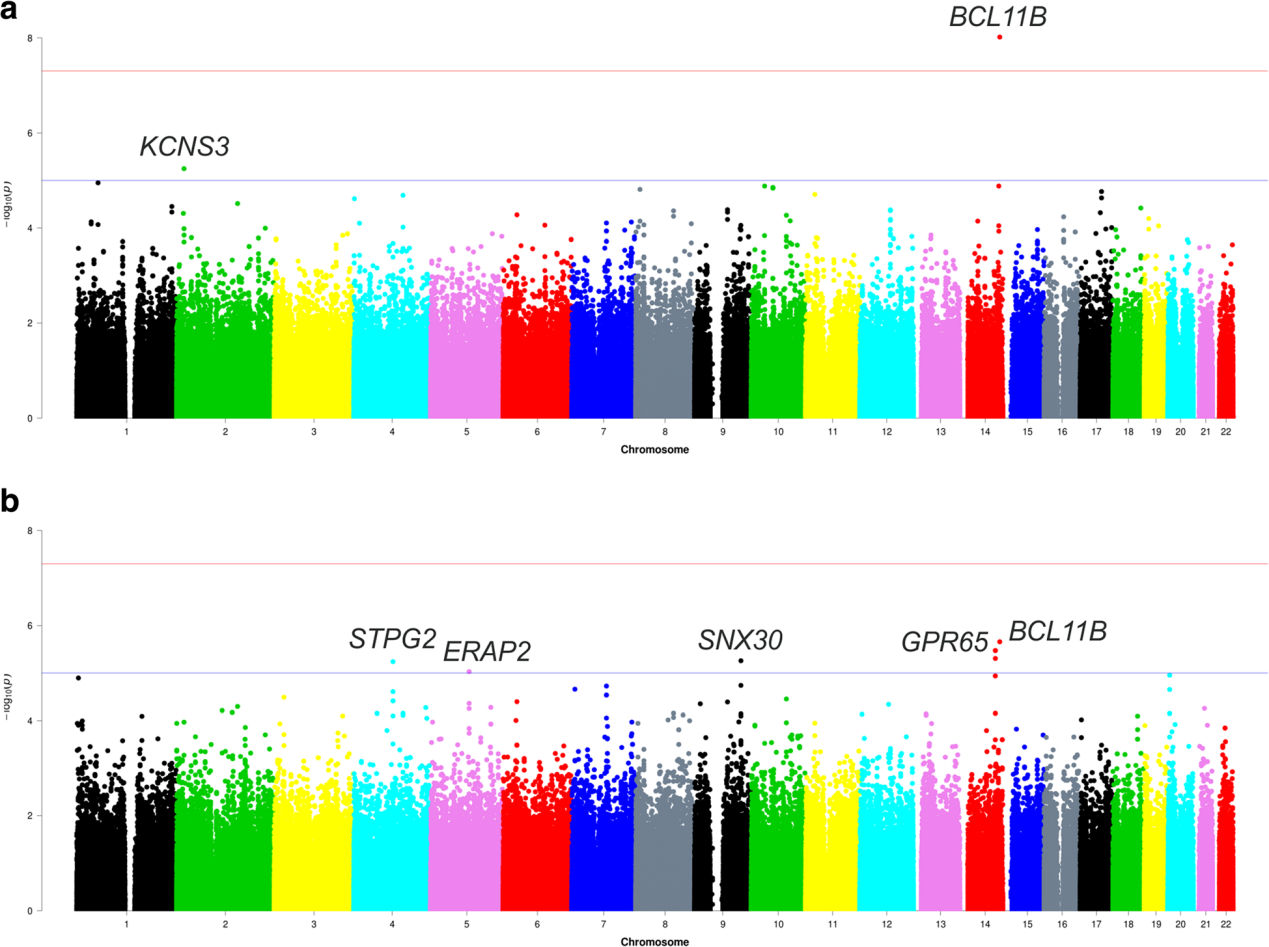
Manhattan plots of the association *P* values from the discovery GWAS in GENRES. (**a**) SBP dipping (**b**) DBP dipping. The y-axis shows the -log_10_(*P* values) of each genotyped SNP and the x-axis shows their chromosomal position. The horizontal lines correspond to genome-wide (5 × 10^−8^) and suggestive (1 × 10^− 5^) *P* value thresholds. SNPs above the suggestive threshold are annotated. Abbreviations: GWAS; genome-wide association study; SBP, systolic blood pressure; DBP, diastolic blood pressure; SNP; single nucleotide polymorphism

The baseline characteristics of GENRES subjects (the discovery sample) are described in Table 1. Ambulatory BP data used for calculation of the mean BP dipping during placebo administration was derived from four (*n* = 173) or three (*n* = 31) recordings. The Manhattan plots of the GWAS results for BP dipping from the discovery cohort are shown in Fig. 1 and the quantile-quantile plots are depicted in Additional file 3: Figure S2. As detailed in Table 2, one locus (rs4905794 at chromosome 14q32.2 near *BCL11B*) reached genome-wide significance threshold for SBP dipping (systolic night-to-day BP ratio *β* = − 4.8%, *P* = 9.6 × 10^− 9^). The association of rs4905794 was very similar for DBP dipping (diastolic night-to-day BP ratio *β* = − 4.3%, *P* = 2.2 × 10^− 6^). Further five potential loci were identified with suggestive significance (*P* < 1 × 10^− 5^) (Table 2). Local Manhattan plots of the best loci are presented in Additional file 4: Figure S3A-F. A total of 99 SNPs in 62 loci had *P* values < 1 × 10^− 4^ for either SBP or DBP dipping.

**Table 1:** Clinical characteristics of the discovery cohort (GENRES) and replication cohorts (DYNAMIC and DILGOM)

Variables	GENRES	DYNAMIC	DILGOM
*n*	204	183	180
Age (years)	50.7 ± 6.3	47.5 ± 11.7	56.3 ± 11.4
Men (%)	100	60	47
Body Mass Index (kg/m^2^)	26.4 ± 2.7	27.1 ± 4.2	26.6 ± 4.4
Waist-hip ratio	0.99 ± 0.05	0.91 ± 0.08	0.91 ± 0.09
Current smokers (%)	15.7	15.3	7.8
Blood pressure levels (mmHg)			
Office measurements			
SBP	152 ± 13	145 ± 18	129 ± 18
DBP	100 ± 7	92 ± 12	79 ± 10
Ambulatory recordings			
Daytime			
SBP	144 ± 10	140 ± 13	128 ± 12
DBP	99 ± 6	88 ± 8	78 ± 8
Nighttime			
SBP	118 ± 11	118 ± 13	112 ± 12
DBP	81 ± 6	71 ± 9	65 ± 8
Nocturnal dipping			
Night-to-day BP ratio (%)			
SBP	81.9 ± 5.3	84.2 ± 5.7	87.8 ± 6.2
DBP	81.9 ± 4.8	80.4 ± 6.4	83.7 ± 8.1
Percentage of non-dippers (%)			
	7	16	32

Non-dipper is defined as night-to-day BP ratio > 90% for either systolic or diastolic blood pressure dipping. Values are presented as mean mean ± standard deviation. *Abbreviations*: *SBP* systolic blood pressure, *DBP* diastolic blood pressure, *BP* blood pressure

**Table 2:** Top SNPs associated with blood pressure dipping (night-to-day blood pressure ratio) in GENRES and replication in DYNAMIC and DILGOM

Discovery GWAS in GENRES									Replication in DYNAMIC					Replication in DILGOM				
SNP	Chr	Gene	EA/ OA	SBP/ DBP	EAF	*n*	*β (%)*	*P*	EAF	*n*	*β (%)*	*P*	Direction	EAF	*n*	*β (%)*	*P*	Direction
rs4905794	14	*BCL11B*	G/A	SBP	0.08	204	−4.8	9.6 × 10^−9^	0.10	183	−0.8	0.4	same	0.11	180	0.9	0.3	opposite
		eQTL		DBP			−4.3	2.2 × 10^−6^			−1.6	0.13	same			1.0	0.4	opposite
rs2119704	14	*GPR65*	A/C	SBP	0.07	204	−2.9	1.7 × 10^−3^	0.06	183	1.4	0.2	opposite	0.06	180	0.9	0.5	opposite
		eQTL		DBP			−4.6	3.4 × 10^−6^			1.1	0.4	opposite			−0.4	0.8	same
rs10817396	9	*SNX30*	G/A	SBP	0.17	204	1.9	2.4 × 10^−3^	0.18	183	0.3	0.7	same	0.20	180	0.3	0.7	same
		intron		DBP			3.1	5.5 × 10^−6^			−0.1	0.9	opposite			0.6	0.6	same
rs16984571	2	*KCNS3*	G/A	SBP	0.15	201	−2.7	5.6 × 10^−6^	0.14	183	−0.8	0.3	same	0.13	180	−1.2	0.2	same
		intron		DBP			−2.5	1.1 × 10^−4^			−0.7	0.4	same			−1.0	0.4	same
rs12509878	4	lncRNA	C/A	SBP	0.34	203	−1.5	9.8 × 10^−4^	0.37	183	1.3	0.03	opposite	0.37	180	−1.4	0.03	same
		intron		DBP			−2.2	5.8 × 10^−6^			1.1	0.11	opposite			−1.6	0.05	same
rs1230361	5	*ERAP2*	A/C	SBP	0.47	204	1.4	1.1 × 10^−3^	0.49	183	0.5	0.4	same	0.45	180	−0.6	0.3	opposite
		intron/eQTL		DBP			2.1	9.3 × 10^−6^			0.1	0.9	same			−0.7	0.3	opposite

SNPs with *P* < 1 × 10^−5^ for SBP or DBP dipping (covariate-adjusted systolic or diastolic night-to-day BP ratio using additive genetic model) in the discovery GWAS (GENRES) are shown in order of significance. Explanation for selection of related candidate gene is shown under gene name. *Abbreviations*: *GWAS* genome-wide association study, *SNP* single nucleotide polymorphism, *Chr* chromosome, *EA* effect allele, *OA* other allele, *SBP* systolic blood pressure, *DBP* diastolic blood pressure, *EAF* effect allele frequency, *eQTL* expression quantitative trait locus

The GENRES subjects received four different antihypertensive monotherapies in a rotational fashion separated by one-month placebo periods (Additional file 1: Figure S1). We also tested if the associations of the statistically most significant SNPs from the six loci with *P* < 1 × 10^− 5^ derived from the placebo periods were similar when dipping data during drug administration was analyzed. The results are shown in Additional file 5: Figure S4A-F. The effects of the SNPs were consistently in the same direction as during placebo periods and the associations remained statistically significant.

### Replication analysis in DYNAMIC and DILGOM

**Fig. 2:**
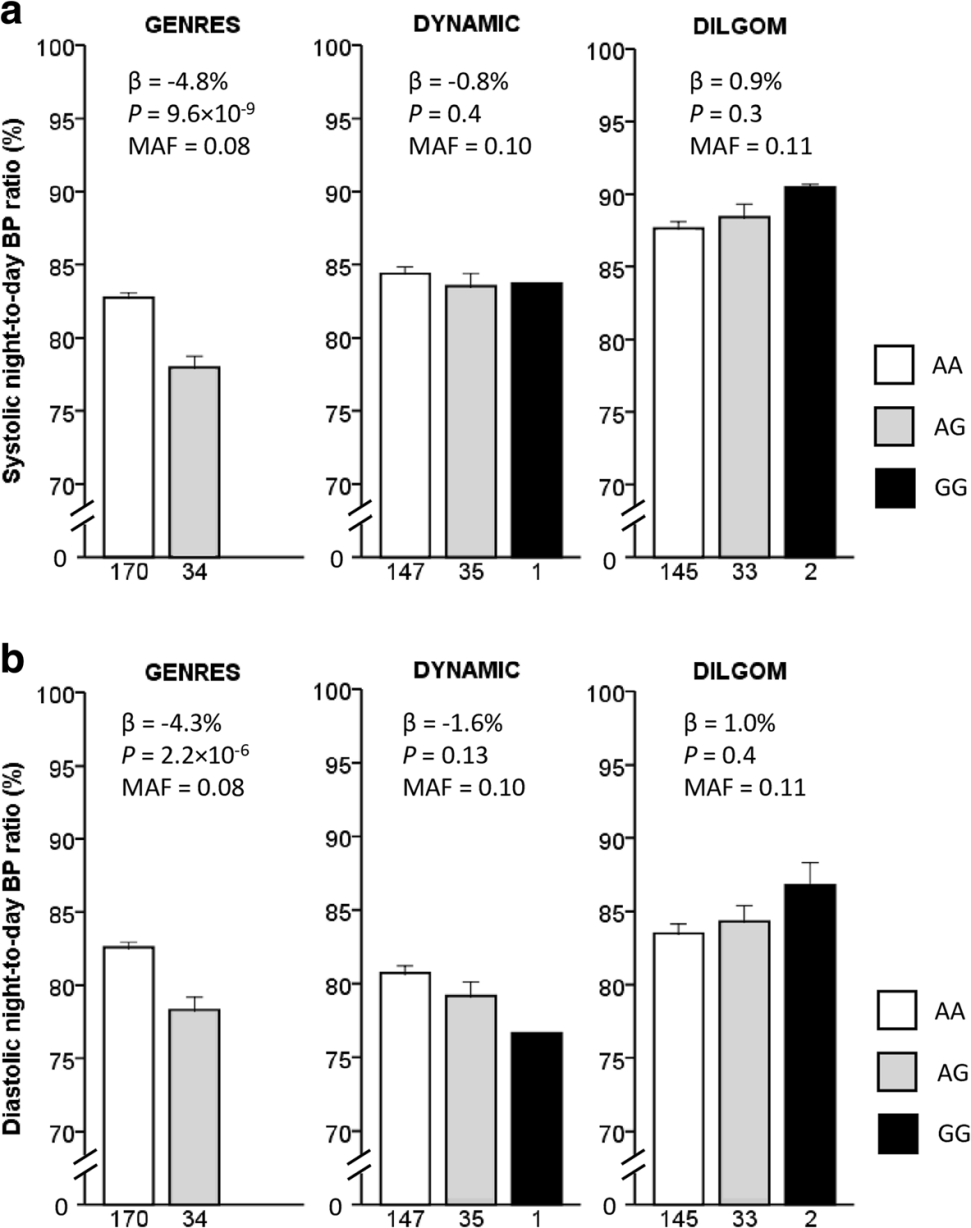
Mean nocturnal blood pressure dipping according to rs4905794 genotypes in the three study samples. (**a**) SBP dipping (systolic night-to-day blood pressure ratio) (**b**) DBP dipping (diastolic night-to-day blood pressure ratio). Error bars indicate standard error of means. Abbreviations: SBP, systolic blood pressure; DBP, diastolic blood pressure

In the replication step, the lead SNPs (most significantly associated SNP) from the genome-wide significant locus and the five loci with *P* values < 1 × 10^− 5^ were followed up in two independent Finnish cohorts, DYNAMIC and DILGOM (Table 1). The majority of the DYNAMIC subjects had elevated blood pressure while DILGOM comprised mostly of normotensive individuals (Table 1). The results of the replication analysis are summarized in Table 2. In DYNAMIC, rs4905794 showing genome-wide significance in GENRES had an effect in the same direction, but the association was not statistically significant (*β* = − 0.8%, *P* = 0.4 for systolic night-to-day BP ratio and *β* = − 1.6%, *P* = 0.13 for diastolic night-to-day BP ratio). The association did not, however, replicate in the population-based DILGOM cohort. Nocturnal BP dipping (night-to-day BP ratio) values according to rs4905794 genotypes in the three study samples are illustrated in Fig. 2. Another SNP, rs16984571 located in *KCNS3* intron, showed effect in the same direction as in GENRES in both replication cohorts, but the associations did not reach statistical significance. In a meta-analysis of all three studies (combined *n* = 567) employing inverse-variance model with fixed effects no SNP showed genome-wide significant *P* values (Additional file 2: Table S2).

### Association of rs4905794 with markers of left ventricular hypertrophy in GENRES

**Fig. 3:**
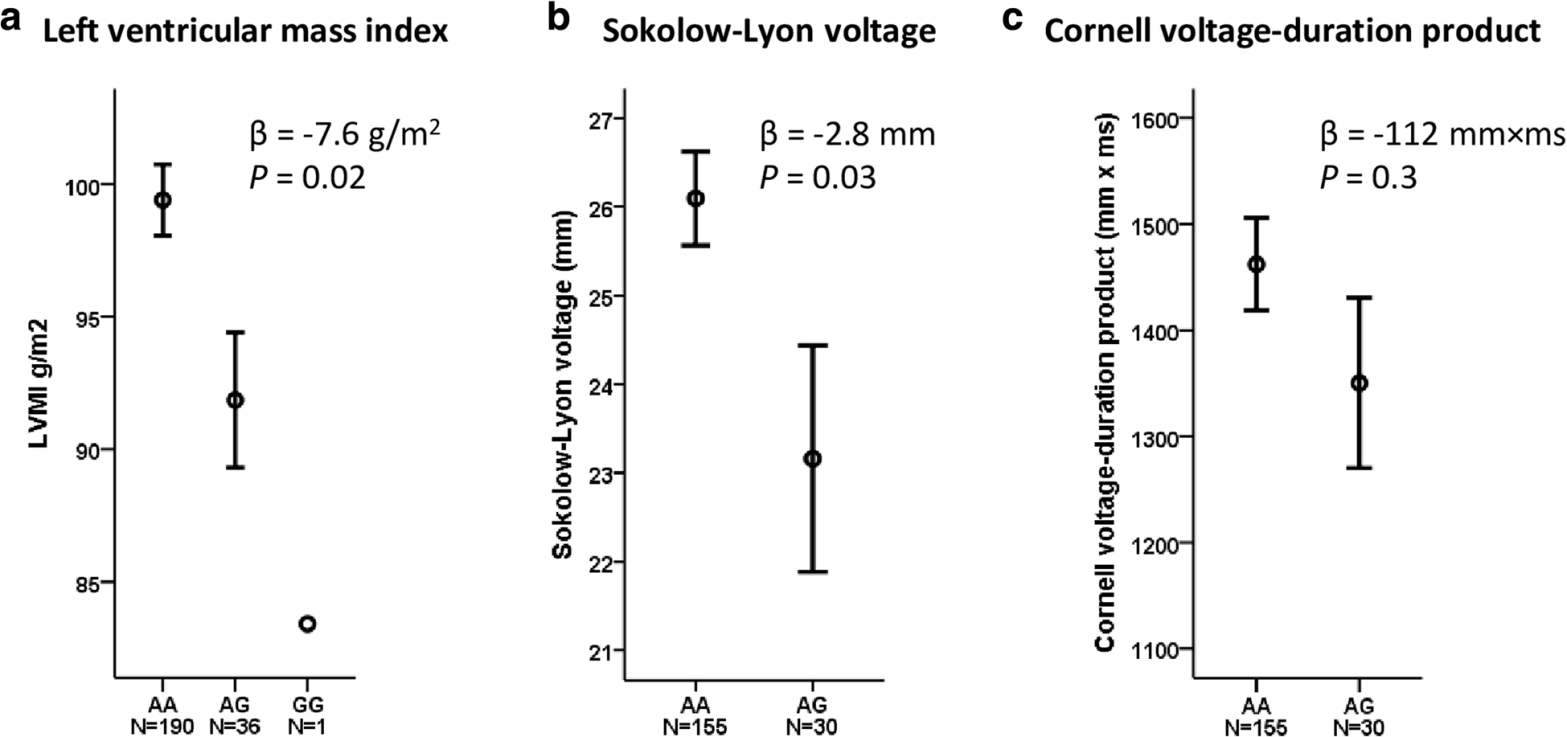
Markers of LVH according to rs4905794 genotypes in GENRES. (**a**) LVMI (*n* = 227), (**b**) Sokolow-Lyon voltage (*n* = 185) (**c**) Cornell voltage-duration product (*n* = 185). Error bars indicate standard error of means. Abbreviations: LVH, left ventricular hypertrophy; LVMI, left ventricular mass index

To explore the possible association of the genome-wide-significant SNP (rs4905794) with markers of cardiovascular target organ damage, we tested for the association of this SNP with indices of LVH in GENRES. Using all GENRES subjects with LVMI and rs4905794 genotype data available (*n* = 227, mean LVMI 98.1 ± 18.5 g/m^2^), rs4905794 was found to significantly associate with LVMI, with the allele associated with larger dipping also associating with smaller left ventricular mass (G allele *β* = − 7.6 g/m^2^, *P* = 0.02) (Fig. 3a). In the 185 GENRES subjects with ECG recordings and rs4905794 genotype available, the mean Sokolow-Lyon voltage was 25.6 ± 6.7 mm and mean Cornell voltage-duration product was 1444 ± 528 mm × ms. The rs4905794 genotype was associated with Sokolow-Lyon voltage (G allele *β* = − 2.8 mm, *P* = 0.03) (Fig. 3b). For Cornell voltage-duration product this association had a tendency to the same direction (G allele *β* = − 112 mm × ms, *P* = 0.3) (Fig. 3c). If BP dipping was included as a covariate in the analyses, the *P* values for the associations remained the same.

### Association of BP dipping with circadian gene polymorphisms

In GENRES GWAS, no SNPs mapping within the transcript boundaries of the selected circadian genes had *P* values < 1 × 10^− 4^ (Additional file 2: Table S3). Using set-based tests to evaluate the combined effect of independent SNPs in each gene, the retinoic acid-related orphan receptor genes (*RORA, RORB, RORC*) had the smallest *P* values (Additional file 2: Table S4). *RORB* had a suggestively significant *P* value 0.03 for association with SBP dipping, but this association did not survive multiple testing. Replication analysis for circadian gene SNPs from two earlier candidate gene studies is summarized in Additional file 2: Table S5. These SNPs did not significantly replicate in our study.

### Replication of previously reported associations of CAD SNPs and BP dipping

In our study, rs9818870 was associated with both systolic and diastolic night-to-day BP ratio (*β* = 1.4%, *P* = 0.03 and *β* = 2.3%, *P* = 0.002, respectively) in consistent direction (Additional file 2: Table S5), thus further supporting the association of this SNP with BP dipping. The associations of the other four CAD SNPs found by Wirtwein et al. [12] did not significantly replicate in our study.

## Discussion

We report here the first GWAS on nocturnal BP dipping. When using a sample of hypertensive patients subjected to repeated (mostly four times) 24-h ABPMs on placebo, a specific SNP (rs4905794) reached genome-wide significance for association with this phenotype, and five additional loci showed *P* values < 1 × 10^− 5^. It is noteworthy that the associations of all six SNPs remained very consistent even when the analyses were conducted during administration of four different antihypertensive drug monotherapies. We sought to replicate the findings in two independent Finnish populations. Compared to GENRES, rs4905794 showed similar, although not statistically significant, association with BP dipping in DYNAMIC, comprising both hyper- and normotensive individuals, but did not replicate in DILGOM, consisting of a population-based sample of individuals. rs4905794 was also associated with LVMI and Sokolow-Lyon voltage in GENRES.

rs4905794 maps to intergenic region on chromosome 14 that is thought to harbor various regulatory features that affect expression of the nearby *BCL11B* gene [37]. In eQTL analysis using publicly available gene expression data sets, rs4905794 was associated with *BCL11B* expression in hippocampus (*n* = 111, *P* = 0.001 in GTEx database [31, 32], Additional file 2: Table S6) and in putamen (*n* = 133, *P* = 0.0001 in Braineac database [38, 39]) with the same direction of effect. rs4905794 has previously been associated with blood lipoprotein(a) level (*P* = 7.6 × 10^− 5^ in NHLBI Family Heart Study [40] data published in database of genotypes and phenotypes (dbGaP) [41, 42]), a well-defined cardiovascular risk factor [43], that has been shown to closely correlate with nighttime BP level and nocturnal BP dipping [44].

*BCL11B* codes for a zinc-finger type transcription factor that has an established role in development and maintenance of central nervous system and regulation of T-cell development (for review, see ref. [45]). However, the role of *BCL11B* in regulation BP is not known. A recent GWAS on carotid-femoral pulse-wave velocity, the standard measurement of aortic stiffness, identified SNPs mapping to the same potential 3′ enhancer region near *BCL11B* at genome-wide significance and the top SNP (rs1381289) was also associated with increased risk for CAD events and heart failure [46]. Interestingly, Cherrier et al. have shown that BCL11B has a protective role in cardiomyocytes by repressing P-TEFb-mediated intercellular signaling that otherwise results in hypertrophic cardiomyopathy in mice [47]. In addition, experimental data supports a role for BCL11B in the direct regulation of circadian rhythm. BCL11B was shown to interact with histone deacetylase 1 (HDAC1) [48], and the nucleosome remodeling and histone deacetylase (NuRD) complex [49]. Both HDAC1, as a complex with SIN3 [50], and the NuRD complex [51] are in turn involved in the regulation of rhythmic expression of mammalian circadian clock PER genes in vitro. In summary, *BCL11B* could affect BP regulation and target organ damage through various mechanisms including the regulation of circadian rhythm, supporting the findings from our GWAS.

Available expression and phenotype databases seem to provide support for the role of some of the lead SNPs reported in the present study (Additional file 2: Tables S6 and S7). Three specific SNPs turned out to be eQTLs for nearby genes. Firstly, there was a strong correlation between intergenic SNP rs1230361 genotype and endoplasmic reticulum aminopeptidase 2 (*ERAP2*) expression in all available GTEx datasets (meta-analysis *P* value 1.0 × 10^− 200^) [31, 32]. In a previous candidate gene study rs2549782 (*r*^*2*^ = 0.40, *D’* = 0.69 with rs1230361 in GENRES) was shown to be associated with pre-eclampsia [52]. Also, in UK Biobank GWAS data [35, 36], rs1230361 was nominally associated with DBP (*P* = 0.002) supporting the role of this SNP in blood pressure regulation, and with two out of seven available sleep traits (getting up in morning, chronotype). Secondly, rs2119704 was an eQTL for *GPR65* expression (meta-analysis *P* value 2.2 × 10^− 10^), and rs10817396 for *SNX30* expression (meta-analysis *P* value 4.1 × 10^− 7^) in selected tissues from the GTEx database [31, 32]. rs10817396 was also associated with both SBP (*P* = 0.03) and DBP (*P* = 0.01) and two out of seven available sleep traits (chronotype, sleeplessness) in UK Biobank GWAS data [35, 36]. It is also noteworthy, that intronic rs16984571 (*KCNS3*), which had an effect in consistent direction in both replication cohorts, also associated with chronotype in UK Biobank GWAS data. *KCNS3* codes for a subunit (Kv9.3) of voltage-gated K^+^ channel that regulates the contraction of arterial smooth muscle [53].

Circadian clock genes have been shown to regulate BP in animal studies [8, 9]. In our GWAS of BP dipping, there were no SNPs showing *P* values < 1 × 10^− 4^ and mapping to circadian genes, and in set-based tests no gene showed enrichment of small *P* values. We could not replicate the positive associations of circadian gene polymorphisms with BP dipping reported by Leu et al. [10] and Sheng et al. [11]. However, the carriers of A allele of rs3816358 of *BMAL1* that associated with non-dipping in both Taiwanese (OR = 1.50, *P* = 0.03) [9] and Chinese (*β* = 3.25%, *P* = 0.04) [10] subjects had reduced SBP (*β* = 0.8%, *P* = 0.2) and DBP (*β* = 1.0%, *P* = 0.18) dipping in GENRES. We attempted to replicate the findings of Wirtwein et al. [12] on five CAD risk loci derived from large GWAS studies that were associated with non-dipping status and found that the A allele of rs9818870 in *MRAS* was significantly associated with reduced SBP and DBP dipping in our hypertensive patients with no evidence of CAD.

The present study has several important limitations. The limited sample size of the discovery cohort (GENRES) and the relatively small sizes of the available replication cohorts constitute an obvious issue. Second, while GENRES and one of the replication samples (DYNAMIC) were dominated by hypertensive individuals, the other (DILGOM) was derived from a population survey. This may have caused a selection bias in GENRES. In addition the definition of awake and sleep period was different in DILGOM compared to GENRES and DYNAMIC. Together, these two issues may have caused smaller effect sizes of the test variable (nocturnal BP dipping), resulting in possible false negative findings in DILGOM. Third, both men and women were included in DYNAMIC and DILGOM, while GENRES consisted of men only. On the other hand, the main strength of our study is the use of repeated (mostly four times) ABPMs during placebo in GENRES. We also believe that acquisition of parallel data under the four different drug monotherapies in GENRES strongly support our data. Even though the results should be interpret with caution due to the small sample size, they encourage further studies using novel well-defined phenotypes to find new genetic associations in human hypertension.

## Conclusions

To our knowledge, our study is the first GWAS conducted on BP dipping in human hypertension. Using a carefully controlled pharmacogenetic study platform, we found a genome-wide significant association between BP dipping and rs4905794 near *BCL11B* gene, with similar tendency in another hypertensive patient cohort; however it was not replicated in a population based sample. rs4905794 was also associated with markers of LVH. In addition, we found some support for the association between BP dipping and rs9818870, previously suggested to associate with nondipping pattern in patients with CAD. In contrast, we did not find evidence that polymorphisms of circadian clock genes would play a marked role in determining BP dipping. Together with previous evidence, our results encourage further research into the role of *BCL11B* in pathophysiology of human hypertension.

## Additional files

Additional file 1: **Figure S1.** The GENRES Study design. (DOC 126 kb)

Additional file 2: **Table S1.** Covariates used for calculation of night-to-day blood pressure ratio residuals in GENRES. **Table S2.** Meta-analysis of blood pressure dipping (night-to-day blood pressure ratio) in all three studies (*n* = 567). **Table S3.** Association of SNPs of circadian genes with blood pressure dipping (night-to-day blood pressure ratio) in GENRES. **Table S4.** Association of circadian genes with blood pressure dipping (night-to-day blood pressure ratio) in GENRES. **Table S5.** Replication of SNPs previously associated with blood pressure dipping in GENRES. **Table S6.** Functional analysis of the lead SNPs associated with blood pressure dipping (night-to-day blood pressure ratio) in GENRES GWAS. **Table S7.** Association of the top blood pressure dipping SNPs with selected UK Biobank GWAS phenotypes. (XLS 167 kb)

Additional file 3: **Figure S2.** Quantile-quantile plots of the genome-wide association results in the discovery cohort (GENRES). (DOC 80 kb)

Additional file 4: **Figure S3A-F.** LocusZoom plots of the top loci for blood pressure dipping. (DOC 591 kb)

Additional file 5: **Figure** **S4A-F.** Systolic and diastolic blood pressure dipping (night-to-day blood pressure ratio) during placebo and drug treatment periods according to top six SNPs genotypes in GENRES. (DOC 457 kb)

## Abbreviations

ABPM: Ambulatory blood pressure measurement
BMI: Body mass index
BP: Blood pressure
CAD: Coronary artery disease
DBP: Diastolic blood pressure
DILGOM: Dietary, lifestyle and genetic determinants of obesity and metabolic syndrome
DYNAMIC: Haemodynamics in primary and secondary hypertension
ECG: Electrocardiogram
eQTL: Expression quantitative trait locus
GENRES: Genetics of drug responsiveness in essential hypertension
GTEx: Genotype tissue expression database
GWAS: Genome-wide association study
LVH: Left ventricular hypertrophy
LVM: Left ventricular mass
LVMI: Left ventricular mass index
PC: Principal component
SBP: Systolic blood pressure
SNP: Single nucleotide polymorphism
