# Supplementary material for: Genome-wide association study of nocturnal blood pressure dipping in hypertensive patients
Source: BMC Med Genet. 2018 Jul 4;19:110. doi: 10.1186/s12881-018-0624-7 (PMC6032801; doi:10.1186/s12881-018-0624-7)
Supplement: Supplementary file 1 — Figure S1. The GENRES Study design. (DOC 126 kb) [file 12881_2018_624_MOESM1_ESM.doc]

**Figure S1** **The GENRES Study design.** The timings of the placebo ABPMs used for the discovery GWAS are marked in the figure with black arrows and blue boxes. Abbreviations: ABPM, ambulatory blood pressure measurement; ECG, electrocardiography

**
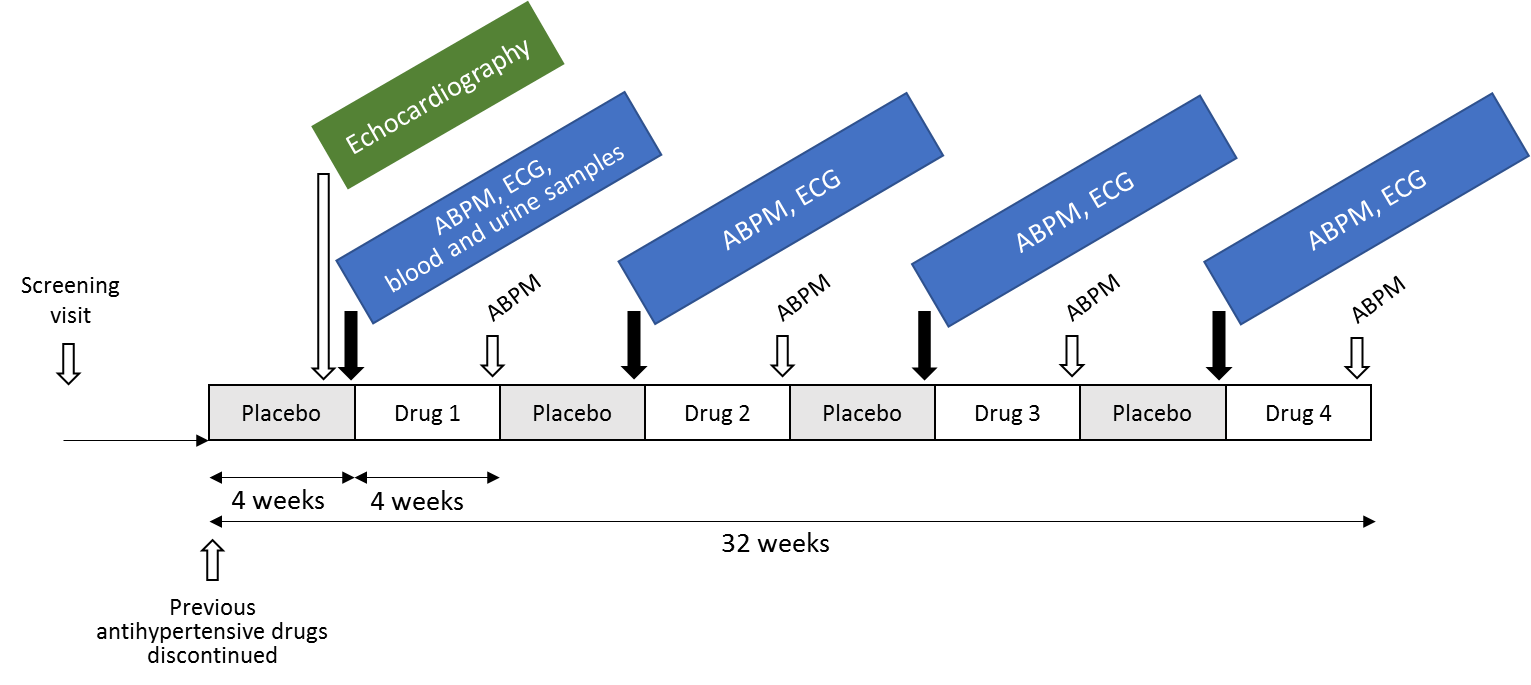
**
