# Supplementary material for: Genome-wide association study of nocturnal blood pressure dipping in hypertensive patients
Source: BMC Med Genet. 2018 Jul 4;19:110. doi: 10.1186/s12881-018-0624-7 (PMC6032801; doi:10.1186/s12881-018-0624-7)

**Figure S2** **Quantile-quantile plots of the genome-wide association results in the discovery cohort (GENRES).** This figure shows the Q-Q plots created in R for two-sided *P* values of the genome-wide association analysis on **(A)** systolic BP dipping (systolic night-to-day BP ratio) and **(B)** diastolic BP dipping (diastolic night‑to‑day BP ratio). The x-axis shows the expected distribution of ­‑log_10_(*P* values) and the y-axis shows the observed ‑log_10_(*P* values). Abbreviations: BP, blood pressure.

**A)**

**
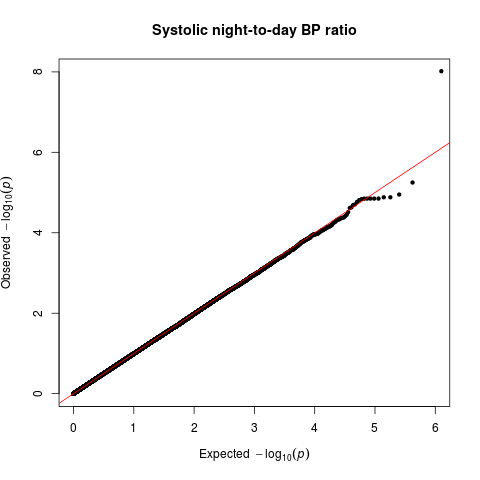
**

**B)**


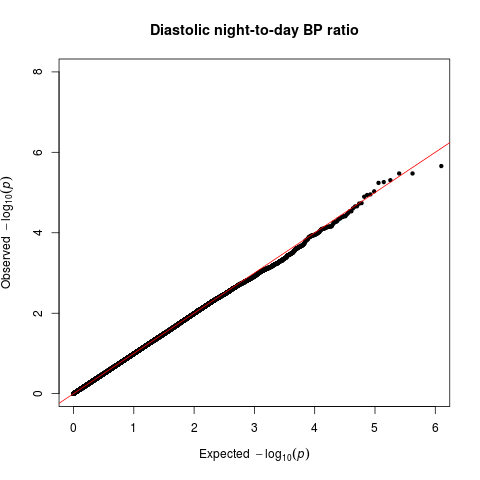

Supplement: Supplementary file 3 — Figure S2. Quantile-quantile plots of the genome-wide association results in the discovery cohort (GENRES). (DOC 80 kb) [file 12881_2018_624_MOESM3_ESM.doc]
