# Supplementary material for: Genome-wide association study of nocturnal blood pressure dipping in hypertensive patients
Source: BMC Med Genet. 2018 Jul 4;19:110. doi: 10.1186/s12881-018-0624-7 (PMC6032801; doi:10.1186/s12881-018-0624-7)

**Figure S3 LocusZoom plots of the top loci for blood pressure dipping (night-to-day blood pressure ratio).** This figure shows the regional Manhattan plots created with LocusZoom for the best loci of blood pressure dipping (night-to-day blood pressure ratio) in the discovery cohort (GENRES). The y-axis shows the ‑log_10_(*P* values) of each genotyped SNP and the x-axis shows their chromosomal position. Blue line depicts the recombination rate from HAPMAP(EU) population. The SNPs are colored to reflect pairwise LD (*r^2^*) with the most significantly associated SNP in GENRES. Genes mapping to each locus are shown in the lower panel. **(A)** rs4905794 (SBP dipping), **(B)** rs2119704 (DBP dipping), **(C)** rs10817396 (DBP dipping), **(D)** rs16984571 (SBP dipping), **(E)** rs12509878 (DBP dipping), **(F)** rs1230361 (DBP dipping). Abbreviations: SNP, single nucleotide polymorphism; LD, linkage disequilibrium; SBP, systolic blood pressure; DBP, diastolic blood pressure.

**A)**


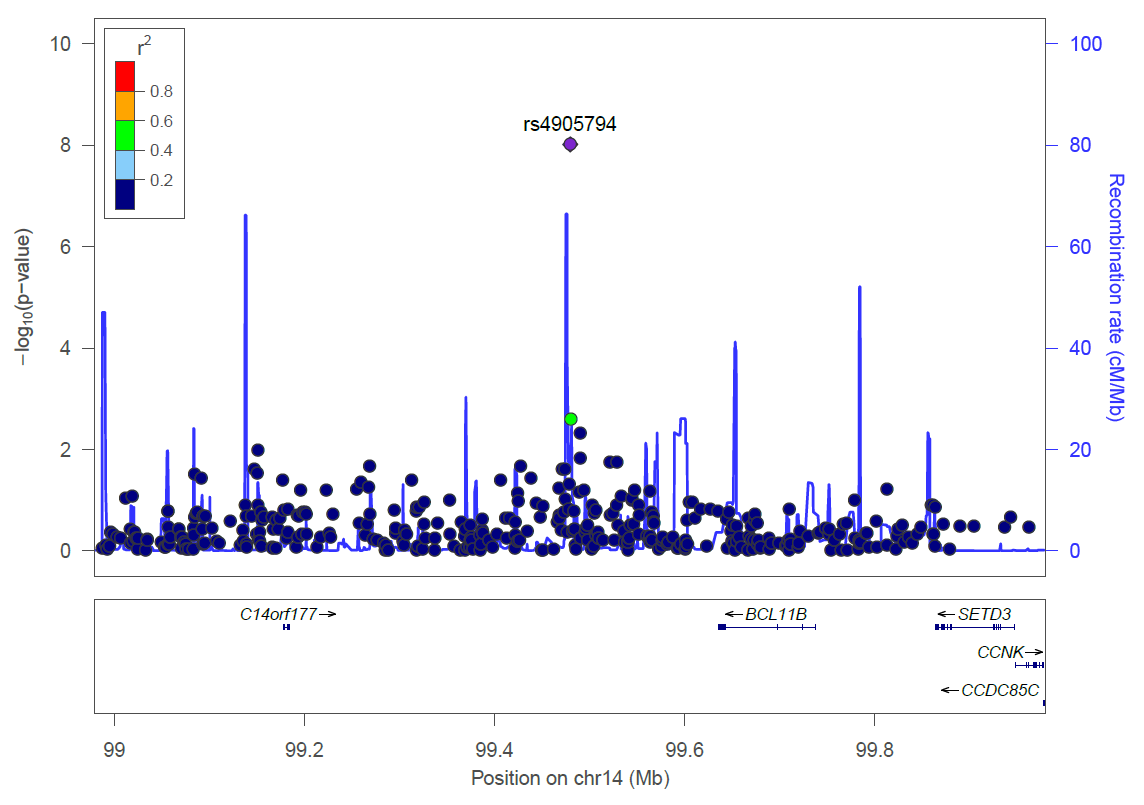


**B)**


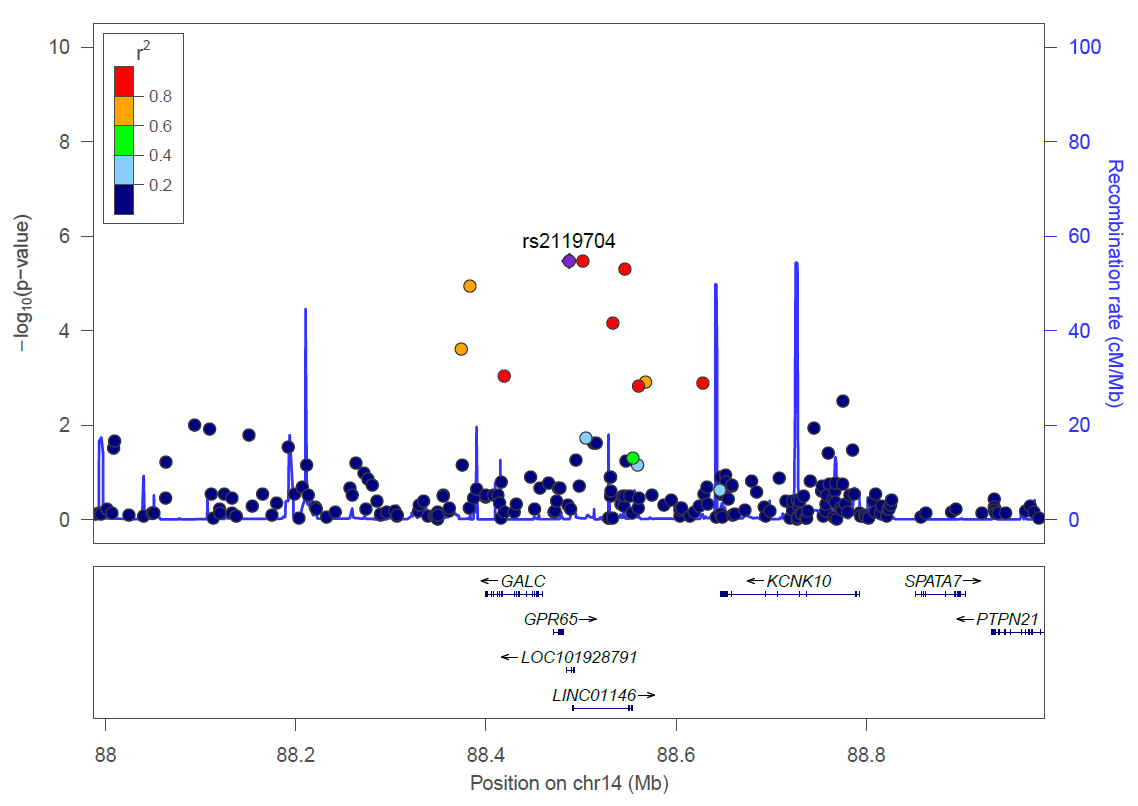


**C)**


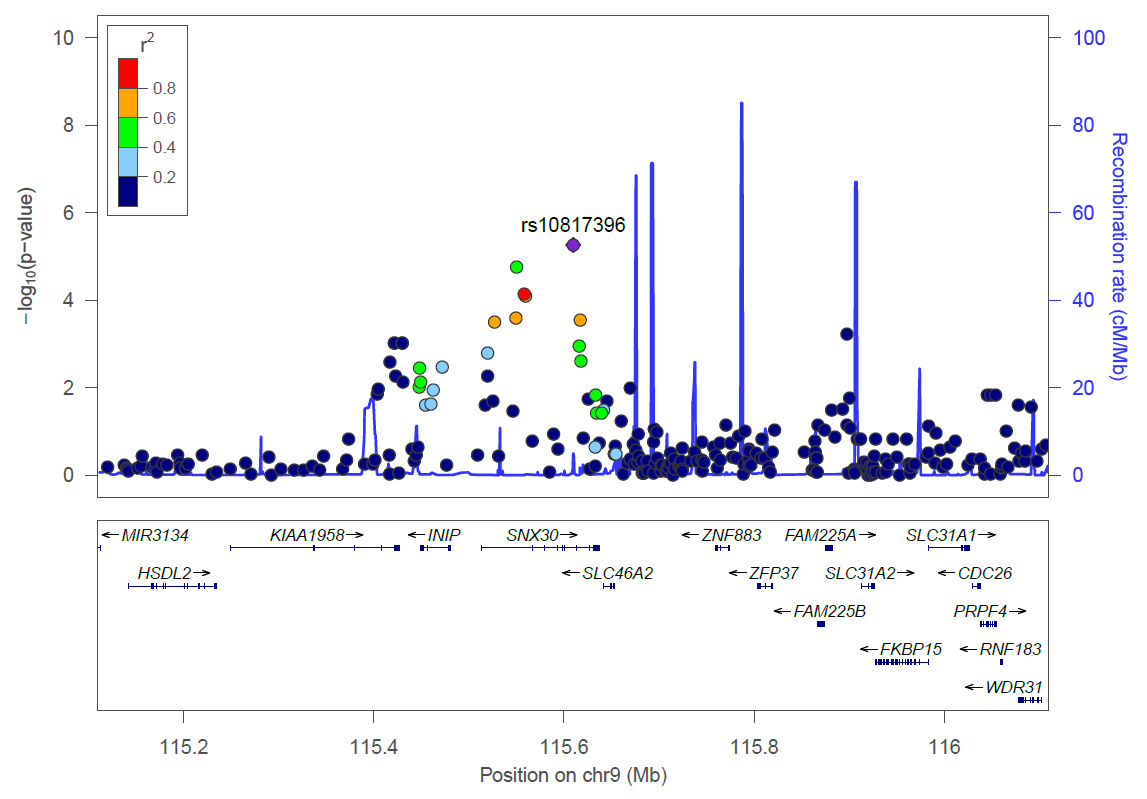


**D)**


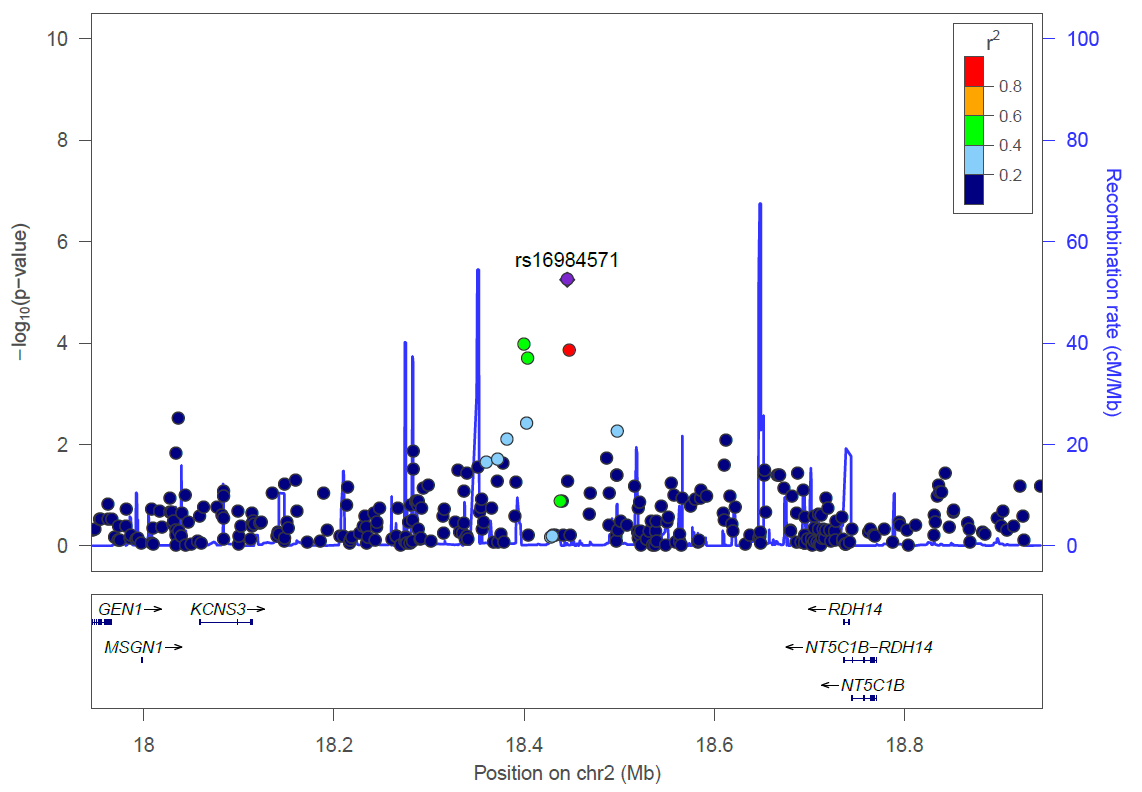


**E)**


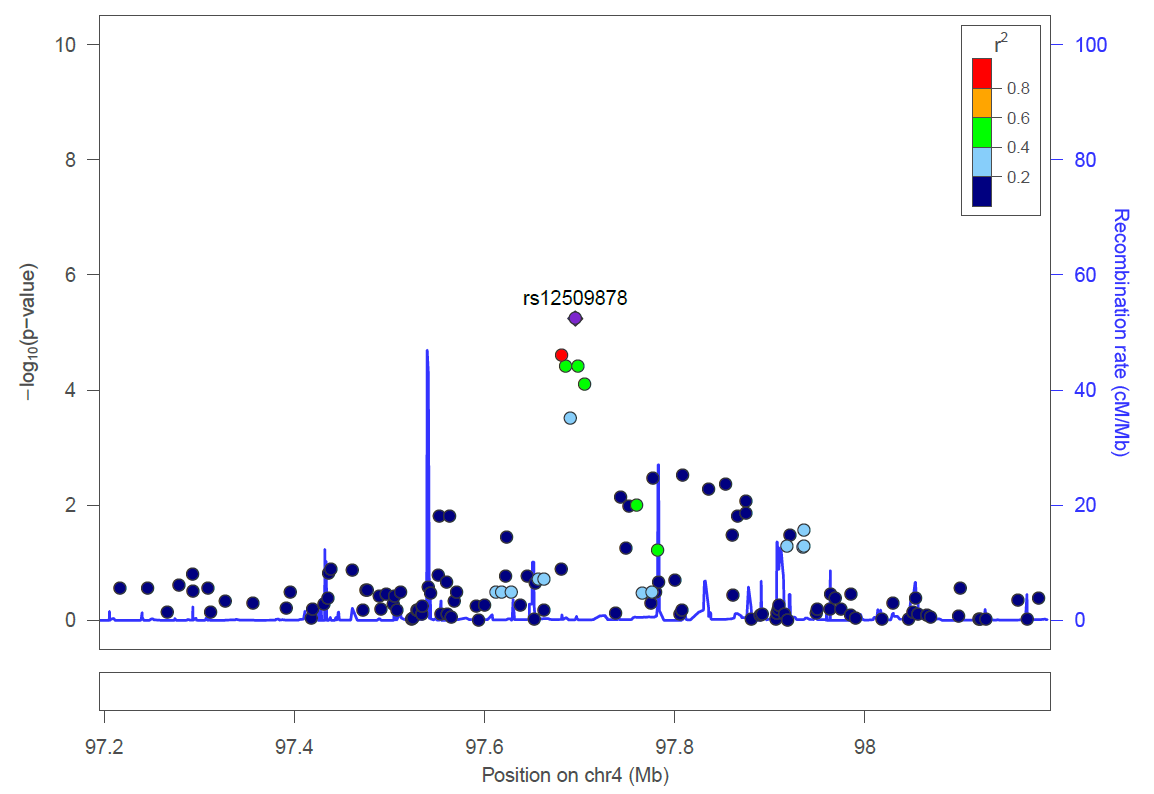


**F)**


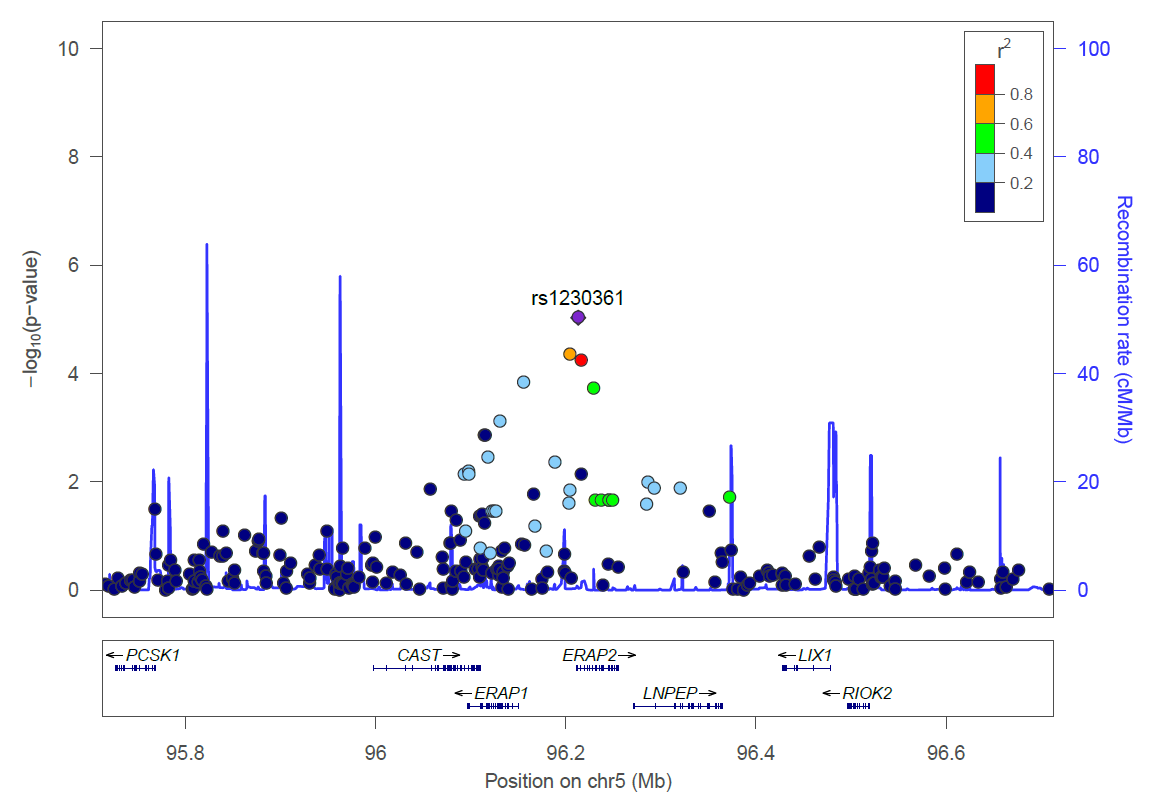

Supplement: Supplementary file 4 — Figure S3A-F. LocusZoom plots of the top loci for blood pressure dipping. (DOC 591 kb) [file 12881_2018_624_MOESM4_ESM.doc]
