# Supplementary material for: Genome-wide association study of nocturnal blood pressure dipping in hypertensive patients
Source: BMC Med Genet. 2018 Jul 4;19:110. doi: 10.1186/s12881-018-0624-7 (PMC6032801; doi:10.1186/s12881-018-0624-7)

**Figure S4A**: Systolic and diastolic blood pressure dipping (night-to-day blood pressure ratio) during placebo and drug treatment periods according to rs4905794 genotypes in GENRES. Night-to-day blood pressure ratio has been adjusted for significant covariates as described in ‘Methods’ section. Error bars indicate standard error of means. Abbreviations: BP, blood pressure.


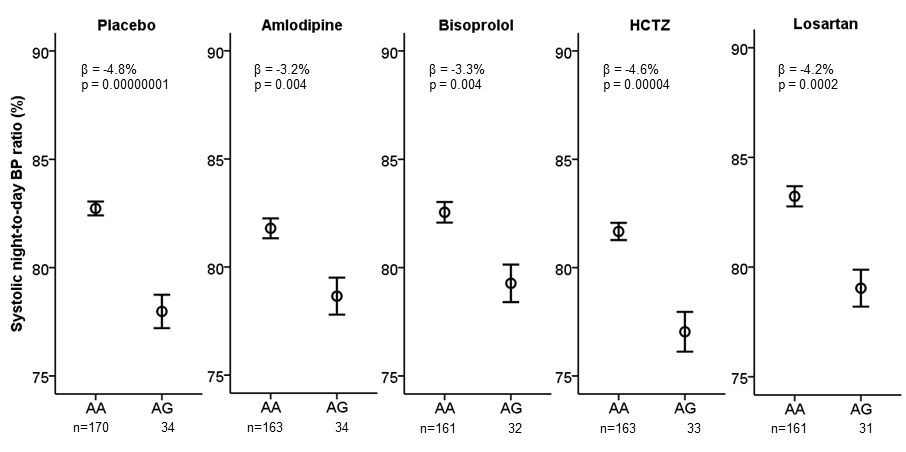


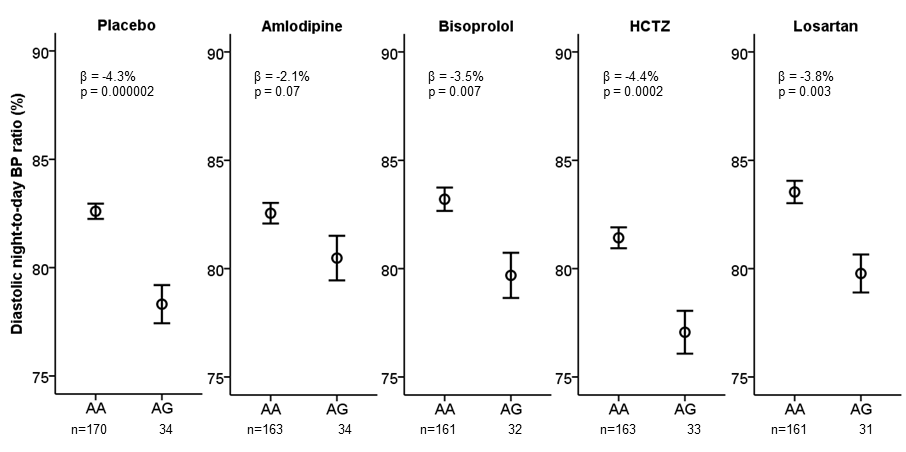


**Figure S4B**: Systolic and diastolic blood pressure dipping (night-to-day blood pressure ratio) during placebo and drug treatment periods according to rs2119704 genotypes in GENRES. Night-to-day blood pressure ratio has been adjusted for significant covariates as described in ‘Methods’ section. Error bars indicate standard error of means. Abbreviations: BP, blood pressure.


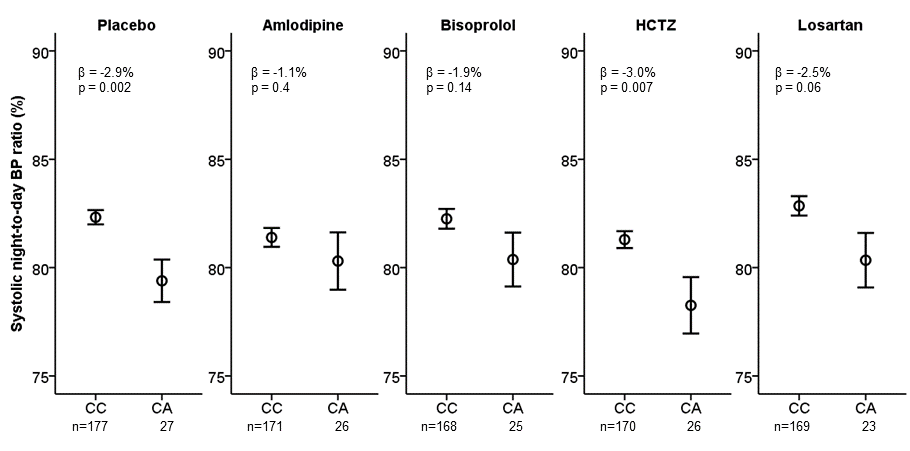


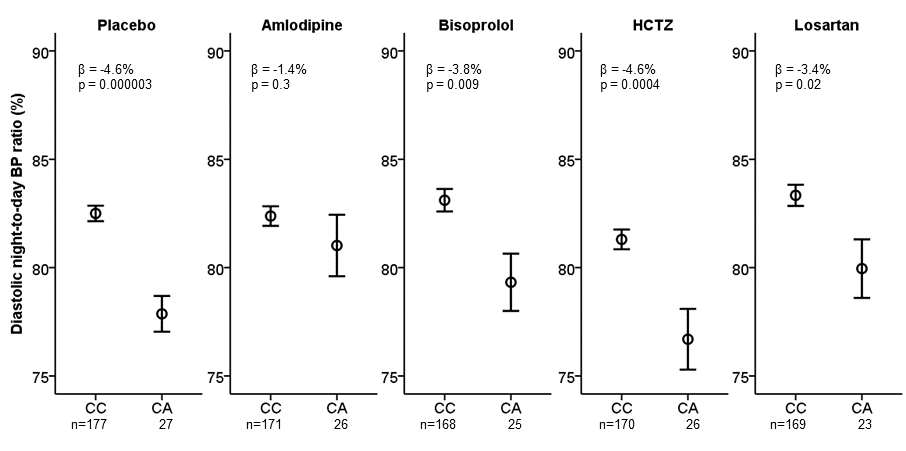


**Figure S4C**: Systolic and diastolic blood pressure dipping (night-to-day blood pressure ratio) during placebo and drug treatment periods according to rs10817369 genotypes in GENRES. Night-to-day blood pressure ratio has been adjusted for significant covariates as described in ‘Methods’ section. Error bars indicate standard error of means. Abbreviations: BP, blood pressure.


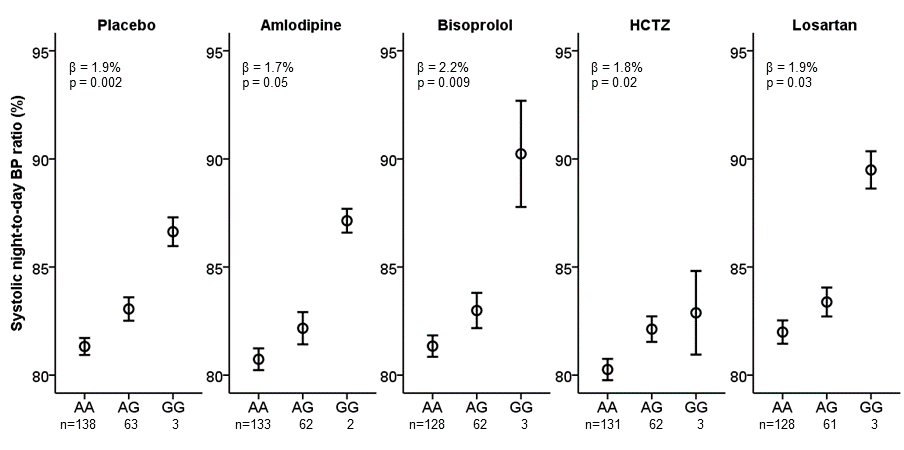


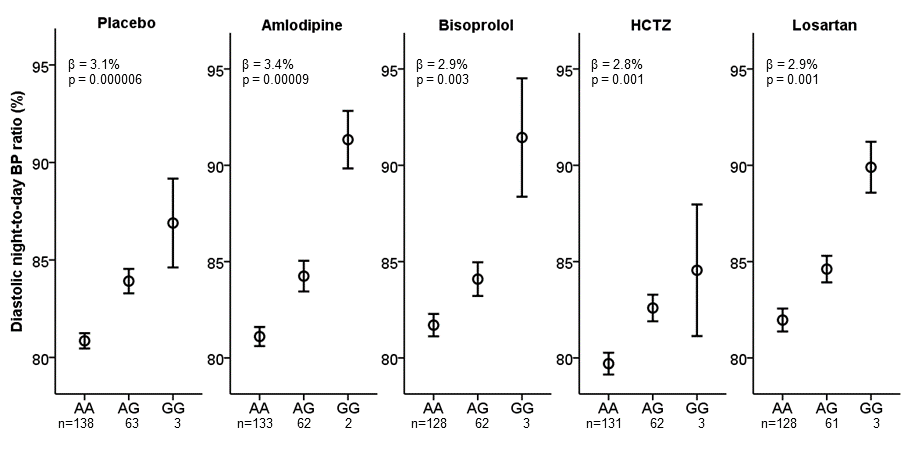


**Figure S4D**: Systolic and diastolic blood pressure dipping (night-to-day blood pressure ratio) during placebo and drug treatment periods according to rs16984571 genotypes in GENRES. Night-to-day blood pressure ratio has been adjusted for significant covariates as described in ‘Methods’ section. Error bars indicate standard error of means. Abbreviations: BP, blood pressure.


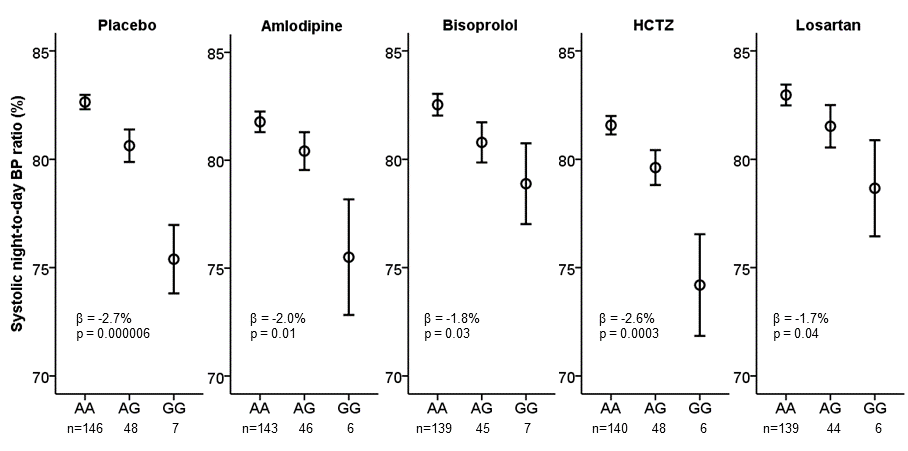


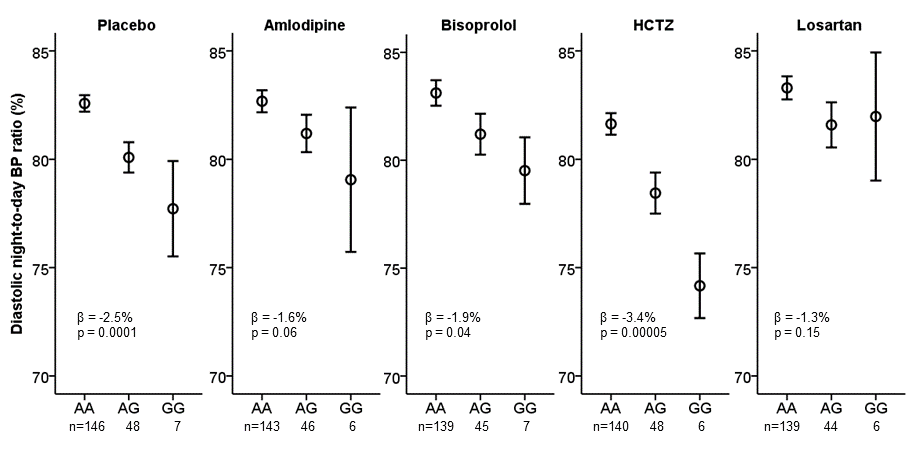


**Figure S4E**: Systolic and diastolic blood pressure dipping (night-to-day blood pressure ratio) during placebo and drug treatment periods according to rs12509878 genotypes in GENRES. Night-to-day blood pressure ratio has been adjusted for significant covariates as described in ‘Methods’ section. Error bars indicate standard error of means. Abbreviations: BP, blood pressure.


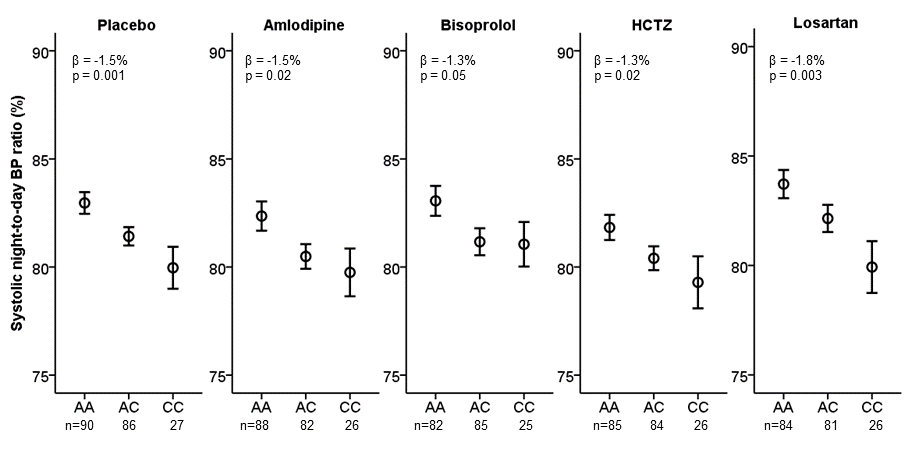


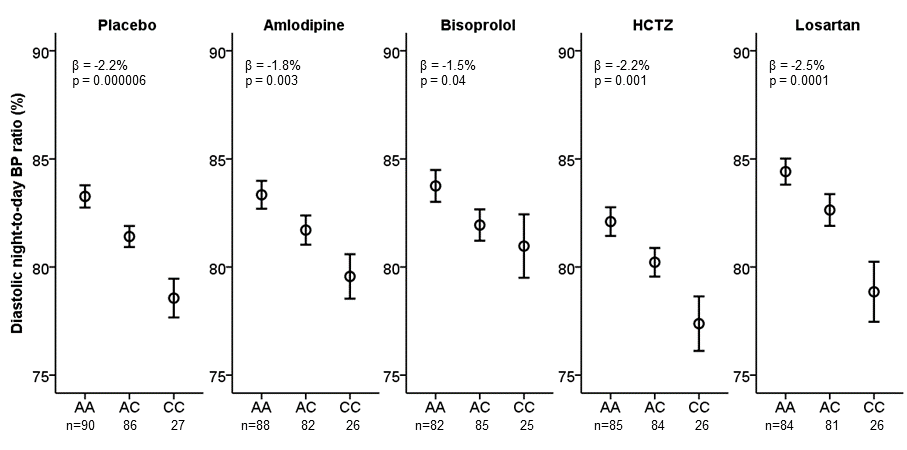


**Figure S4F**: Systolic and diastolic blood pressure dipping (night-to-day blood pressure ratio) during placebo and drug treatment periods according to rs1230361 genotypes in GENRES. Night-to-day blood pressure ratio has been adjusted for significant covariates as described in ‘Methods’ section. Error bars indicate standard error of means. Abbreviations: BP, blood pressure.


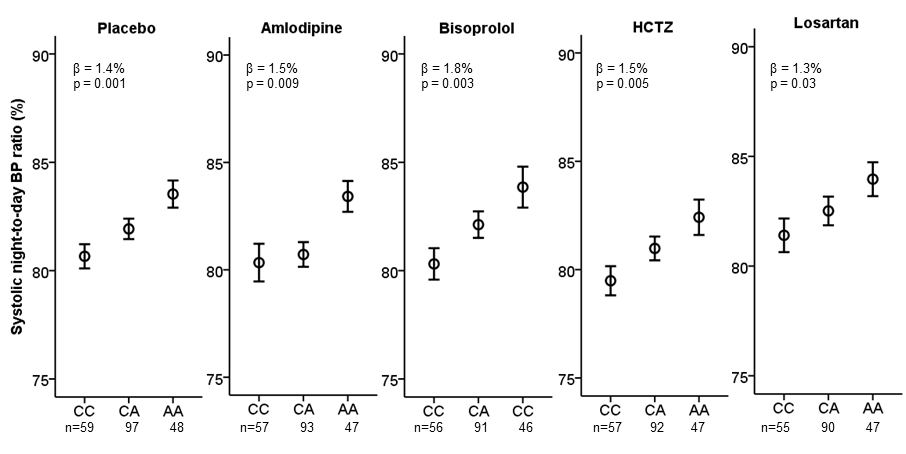


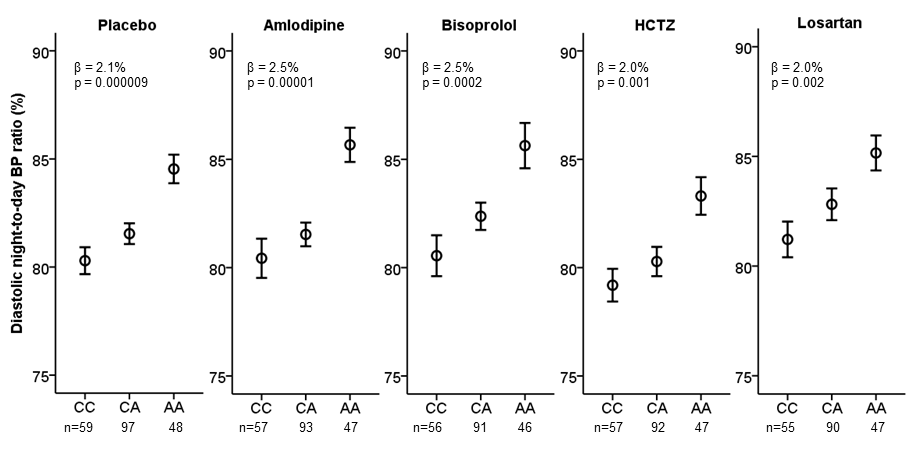

Supplement: Supplementary file 5 — Figure S4A-F. Systolic and diastolic blood pressure dipping (night-to-day blood pressure ratio) during placebo and drug treatment periods according to top six SNPs genotypes in GENRES. (DOC 457 kb) [file 12881_2018_624_MOESM5_ESM.doc]
